# Immediate Postoperative Pain: An Atypical Presentation of Dropped Gallstones after Laparoscopic Cholecystectomy

**DOI:** 10.1155/2015/930450

**Published:** 2015-01-14

**Authors:** Samba Binagi, Jason Keune, Michael Awad

**Affiliations:** ^1^Department of Surgery, Washington University School of Medicine, Campus Box 8109, 660 South Euclid Avenue, St. Louis, MO 63110, USA; ^2^Saint Louis University School of Medicine, St. Louis, MO, USA

## Abstract

Cholecystectomy is one of the most commonly performed surgical procedures in the United States. A common complication is dropped gallstones, and the diversity of their presentation poses a substantial diagnostic challenge. We report the case of a 58-year-old man presenting with chronic right upper quadrant hours status post cholecystectomy. Imaging demonstrated retained gallstones in the perihepatic space and symptoms remitted following their removal via laparoscopic operation. Gallstones are lost in roughly 1 in 40 cholecystectomies and are usually asymptomatic. The most common presentations are months or years status post cholecystectomy due to fistula, abscess, or sinus tract formation. We report this case hoping to bring light to a rare presentation for dropped gallstones and provide advice on the management of this common complication of cholecystectomy.

## 1. Introduction

Laparoscopic cholecystectomy is among the most frequently performed surgical procedures in the United States. A common complication of cholecystectomy is the retention of dropped gallstones in the abdomen. Loose stones are most often a result of gallbladder perforation during dissection or exposure, which occurs in 30% of laparoscopic cholecystectomies. Gallstones are dropped by surgeons in an additional 7% of cholecystectomies and studies suggest that 16 to 50% of dropped stones are unrecovered [1, 2]. In such cases, typical practice is to remove stones intraoperatively. Male gender, old age, obesity, and omental adhesions are risk factors for stone spillage [3]. Because of the limitations of laparoscopic approach, however, stones remain loose in the abdomen after an estimated 2.4% of laparoscopic cholecystectomies [4, 5]. It is estimated that 8.5% of these stones cause complications, leading to complications from dropped gallstones in approximately 2 in 1000 cholecystectomies [6].

Gallstones retained in the abdomen become niduses for inflammation that can lead to development of fistulas, abscesses, or sinus tracts. Stones thus cause a variety of different symptoms, most often presenting as a localized pain either in the perihepatic space or at port sites, probably because stones are most often spilled during dissection of the gallbladder or manipulation of the organ during removal through port sites. While the most commonly reported complication is intra-abdominal abscess, stones can find their way anywhere in the intraperitoneal space and cause a wide array of presentations. Fistulas and abscesses from retained gallstones occur throughout the abdomen [6–8]. Erosion through soft tissue has led to lung infections, stone expectorations, and intragluteal and ovarian abscesses [9–12].

Though uncommon, complications from dropped gallstones can be agonizing to patients and confounding for physicians. In this paper, we present a patient who suffered abdominal pain from retained stones hours after surgery. To our knowledge, this is the first reported case of symptoms from dropped gallstones in the immediate postoperative period. We also explore the diagnostic challenges that his case and similar cases present. Lastly, we discuss measures to reduce the incidence of gallstone retention and hasten the diagnosis and treatment of subsequent complications.

## 2. Case Presentation

The patient is a 58-year-old obese male whose past medical history is significant for laparoscopic cholecystectomy in 2012, nephrolithiasis, spinal osteoarthritis, and exploratory laparotomy for a gunshot wound and bullet fragments who presented with right flank pain since cholecystectomy. The pain was continuous but waxed and waned, reaching levels the patient described at times as eight out of ten on a ten-point Likert scale. He had no prior episodes of such pain. He received some relief from naproxen and hydrocodone/acetaminophen that he takes for osteoarthritis. He denied that the pain interferes with daily life but has been on leave from his job as a construction worker due to chronic osteoarthritic back pain. He denied nausea, vomiting, diarrhea, dysuria, hematuria, dyschezia, and bloody or tarry stool.

The patient's medical history includes hypercholesterolemia, hyperlipidemia, heart palpitations, anxiety, and headaches. His regular medications are aspirin, fenofibrate, gabapentin, and esomeprazole. He denies alcohol use but smokes twenty small cigars per week and marijuana regularly.

His physical examination was notable for right flank tenderness without rebound. Additionally, he had a painless, reducible bulge in his left flank suggestive of a port site hernia from his prior laparoscopy.

Laboratory studies showed bilirubin, liver function tests, and alkaline phosphatase levels within normal limits. Urinalysis showed no hematuria or abnormalities. Computed tomography scanning from an outside institution revealed hyperintensities between the liver and right abdominal wall with surrounding streakiness suggestive of inflammation. Ultrasound of the biliary tree showed no distension or retained gallstones.

**Figure 1:**
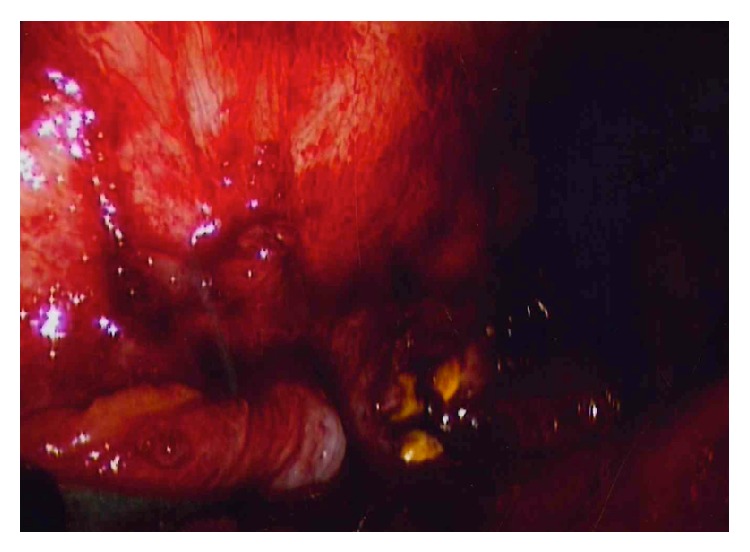
Stones in perihepatic space.

Diagnostic considerations included irritation from dropped gallstones in the perihepatic space, nephrolithiasis, and somatization (Figure 1).

Hypothesizing that the stones were the cause of the patient's pain, we performed an explorative laparoscopy to retrieve the stones and a left flank incisional hernia repair. The patient had extensive omental adhesions to the abdominal wall. Exposure of the lateral edge of the right liver revealed several yellow gallstones. We removed every stone that was visualized intraoperatively; they were 4.4 cubic cm in aggregate volume. The patient reported reduction of right upper quadrant pain in the immediate postoperative period and at a postoperative visit three weeks later stated that his pain had almost completely resolved.

## 3. Discussion

This case may demonstrate a novel presentation for retained gallstones following laparoscopic cholecystectomy. The patient's complaint of several months of sharp, constant pain beginning in the immediate postoperative period is unusual. As Zehetner et al. discussed in their review of complications from dropped gallstones, the most common complications are abscesses, fistulas, and sinus tracts, which typically take weeks to years to present [6].

This patient's male gender, old age, obesity, and adhesions from prior laparotomy put him at increased risk for complications from dropped gallstones [3]. The nonspecific nature of his right upper quadrant pain called to mind a broad differential diagnosis. Due to his age and complicated medical and mental health and surgical history, it required consideration of biliary, hepatic, renal, musculoskeletal, and somatic causes of pain.

The patient endured pain for months and was subjected to a battery of tests. His angst and discomfort could have been minimized had he known that he had loose intraperitoneal gallstones. While it is unclear whether his cholecystectomy surgeon knew about the dropped stones, his experience highlights the importance of clear and complete disclosure to patients.

This case also illustrates the limitations of imaging when it comes to dropped gallstones. As Ramamurthy et al. report, the variety and relative rarity of patients presenting with dropped gallstones, patients' unawareness of loose intraperitoneal stones, and the nondistinctive hyperintensity appearance of gallstones on radiographs often lead radiologists to mistake them for neoplastic deposits, colonic diverticula, or other pathologies [7]. Moreover, gallstones within abscesses are often missed, leading to medical management with antibiotics when surgical removal is indicated and putting patients at risk for reinfection and sepsis [13, 14].

The immediate remission of the patient's pain following laparoscopic removal of remnant gallstones strongly supports our hypothesis that their presence was driving his symptoms. His continued relief at follow-up three weeks postoperatively is further confirmation. The small amount of minor pain that he reported was likely due to persistent inflammation. It could be from a small stone embedded in the abdominal wall, though, so suspicion for abscess or fistula is warranted should his pain persist and worsen or should he develop a fever [7]. That he is vulnerable to subsequent complications from dropped gallstones despite interventions thus far serves as a reminder to physicians that dropped gallstones can cause symptoms at any time in the postoperative period and that prophylactic measures against spillage are the best measure against harmful sequelae [6].

## 4. Conclusions/Recommendations

Patients with acute or severe cholecystitis are more likely to suffer rupture, so strongly urging patients to undergo cholecystectomy when cholecystitis is discovered is encouraged. Though evidence is unclear regarding the expertise of the surgeon and rates of cholecystectomy complications, in acute settings and cases of extreme inflammation where rupture is more likely a more experienced surgeon may reduce risk of complications, including dropped stones [15, 16]. As gallbladder rupture is often the cause of stones being dropped, removal of the intact bladder is the best strategy to prevent dropped stones. Care to minimize use of sharp instruments and electrocautery near inflamed gallbladders may reduce incidence of surgical rupture. Use of an endobag during removal may lower the risk of dropping stones during this critical step [17]. Of course, retrieving stones when they are dropped should reduce the incidence of complications, but conversion to laparotomy for gallstone retrieval is seen as unnecessary [6, 7].

When stones cannot be retrieved following a drop or if it is unclear whether stones remain, a CT scan to rule out the presence of loose stones in the abdomen may be helpful. Operative records and postoperative patient debriefing should reflect the spillage of stones and whether attempts to retrieve them were successful. While loose stones are usually harmless and patient anxiety can lead to unnecessary clinical visits, patient autonomy and safety obligate their full disclosure. There is a dearth of evidence, but frequent follow-up and surveillance have not been shown beneficial in documented cases of dropped stones [3].

Given the diversity of complications dropped gallstones can present, plans for their management must be considered on a case-by-case basis considering the anatomy and severity of the problem along with the health and wishes of the patient. Removal of harmful retained stones with appropriate medical or surgical treatment for complications is generally recommended.
